# The Effect of Antimicrobial Agents on Implant Surface Wettability Changes: An In-Vitro Study

**DOI:** 10.3290/j.ohpd.c_2039

**Published:** 2025-06-03

**Authors:** Rachel Swed, / Elina Nourmand, / Nina K. Anderson, / Rafael Delgado-Ruiz, / Georgios Romanos

**Affiliations:** a Rachel Swed Dental Student, Department of Periodontics and Endodontics, Stony Brook School of Dental Medicine, Stony Brook, NY, USA. Methodology, investigation, resources, data curation, wrote original draft, supervision, read and agreed to the published version of the manuscript.; b Elina Nourmand Dental Student, Department of Periodontics and Endodontics, Stony Brook School of Dental Medicine, Stony Brook, NY, USA. Validation, reviewed and edited the manuscript, read and agreed to the published version of the manuscript.; c Nina K. Anderson Clinical Assistant Professor, Department of Oral Biology and Pathology, Stony Brook School of Dental Medicine, Stony Brook, NY, USA. Software, formal analysis, read and agreed to the published version of the manuscript.; d Rafael Delgado-Ruiz Associate Professor, Department of Prosthodontics and Digital Technology, Stony Brook School of Dental Medicine, Stony Brook, NY, USA. Reviewed and edited the manuscript, read and agreed to the published version of the manuscript.; e Georgios Romanos Professor, Department of Periodontics and Endodontics, Stony Brook School of Dental Medicine, Stony Brook, NY, USA. Conceptualisation, methodology, project administration, funding acquisition, read and agreed to the published version of the manuscript.

**Keywords:** chlorhexidine, herbal extract rinse, implants, wettability.

## Abstract

**Purpose:**

The goal of this in-vitro study was to determine the impact of the antimicrobial disinfecting agents chlorhexidine (Peridex) and an herbal extract (StellaLife) on the wettability of four implant surfaces: titanium machined (TM), titanium-SLA (SLA), titanium alloy (TA), and zirconia.

**Materials and Methods:**

Each implant surface in the form of a disk was disinfected with 0.12% chlorhexidine (Peridex, group 1), peppermint-flavoured herbal extract (StellaLife, group 2), and saline solution as the control liquid (group 3). Using a calibrated micro-syringe, 7.5 µl of each liquid were dispensed on the center of each disk (n = 180). Then, a goniometer was used to measure contact angles between the droplet and the disk surface to evaluate the wettability (hydrophilicity) of each implant surface. The mean from 20 contact angle measurements per liquid and implant surface was calculated. Comparative statistical analysis was performed with ANOVA and Bonferroni correction at the p < 0.05 level of significance.

**Results:**

The Bonferroni post-hoc comparison revealed a statistically significant difference with improved wettability for group 2 compared to groups 1 and 3 for rough-surfaced titanium-SLA implant surfaces.

**Conclusion:**

Overall, titanium implants may have improved hydrophilicity when rinsed with herbal extract antimicrobial agents compared to chlorhexidine.

Dental implant placement has become a routine procedure to replace missing teeth in partially edentulous patients.^
3
^ The wettability of an implant surface is crucial, as it can determine the biological response and events at the bone-implant interface. Wettability can vary based on the surface characteristics of an implant, such as its chemistry, topography, and roughness.^
10
^ Furthermore, the surface wettability (hydrophilicity) of an implant can affect bacterial adhesion, biofilm formation, and most importantly, the rate of osseointegration.^
2
^ Osseointegration is important in implant success, as it is defined as the deposition of bone onto biomaterial devices to anchor the dental prostheses.^
8
^ Hydrophilic surfaces help the early stages of cell adhesion, proliferation, differentiation, and bone mineralisation; therefore, an understanding of wettability and the mechanisms by which it controls an implant’s biological environment can enhance the design of implants to ensure successful outcomes.^
6
^


To calculate the wettability of an implant, the contact angle (CA) between the liquid’s surface and the implant’s surface is measured using a tangent line. The CA can range from 0 to 180°s based on the implant surface and liquid used. When the CA of water is less than 90°s, implant surfaces are characterised as hydrophilic, and conversely, when the contact angle is above 90°s, they are classified as hydrophobic.^
6
^


Implants can be manufactured using different materials. The present study focused on titanium-SLA (SLA), titanium machined (TM), titanium alloy (TA), and zirconia surfaces. Titanium implants have been used for decades due to their favourable properties of biocompatibility, corrosion resistance, and osseointegration ability.^
7
^ Titanium-SLA implant surfaces are treated to increase surface roughness by sandblasting with large-grit particles and then acid etched with sulfuric, hydrochloric, or nitric acid. They provide a favourable biological space for cell migration, differentiation, and attachment, which induces the proliferation and growth of osteoblasts.^
16
^ However, use of titanium implants has increased concerns of the negative effects of titanium particle release within the oral cavity.^
7
^ While such concerns persist, there is insufficient evidence to support a unidirectional causative role of titanium particles as non–plaque-related factors in the etiology of peri-implantitis.^
1
^ Zirconia implants are an alternative biocompatible option which provide histological osseointegration results similar to those of titanium-SLA implants, due to osteoblast attachment to the zirconia surfaces.^
12
^ Zirconia implants have been found to decrease bacterial adhesion and reduce inflammation compared to titanium implants.^
5
^ In addition to titanium-SLA, both commercially pure titanium and titanium alloys are used due to their bioinert properties and cost-effectiveness.^
14
^ Titanium alloy implants, known as Ti-6Al-4V implants, are strong and corrosion resistant thanks to their composition of titanium, 6% aluminum, and 4% vanadium.

Chlorhexidine (CHX) is commonly recommended for biofilm management and infection control before and after implant surgery. When patients use CHX after implant surgery, there is a significant reduction in plaque accumulation and bleeding.^
15
^ Nevertheless, CHX does have several side effects, such as discolouration of teeth, dry mouth, and cytotoxic effects on human cells. Alternatively, an herbal extract rinse (StellaLife) exists which can also minimise inflammation and plaque accumulation without the side effects of CHX. Additionally, it has an analgesic effect and increases fibroblastic activity to help recovery.^
4
^ This research article hypothesises that none of the four implant surfaces will demonstrate statistically significant difference in wettability when disinfected with either CHX or herbal extract rinse.

## MATERIALS AND METHODS

Four implant surfaces in the form of disks were studied: titanium-SLA, titanium alloy, zirconia, and titanium machined. Each implant surface’s CA was measured after being disinfected with each of the three liquids: 0.12% chlorhexidine gluconate, known as Peridex (group 1), an herbal extract, known as StellaLife with peppermint flavor (group 2), and saline solution (group 3). The saline solution was used as the control group while the other two groups served as experimental groups. A total of twelve disks were used for this experiment. Each group used four disks with each disk being a different material. A calibrated micro-syringe was used to dispense 7.5 µl of each liquid on the center of each disk. The CA between the liquid’s surface and the implant’s disk surfaces was measured using a goniometer (Ossila; Sheffield, UK) to gather 20 measurements per liquid and disk. In total, 240 measurements were recorded to evaluate the surface hydrophilicity. Subsequently, a mean was calculated for each liquid and disk, resulting in 12 measurements. Comparative statistical analysis with ANOVA and Bonferroni correction at the p < 0.05 level was performed.

## RESULTS

**Fig 1 fig1:**
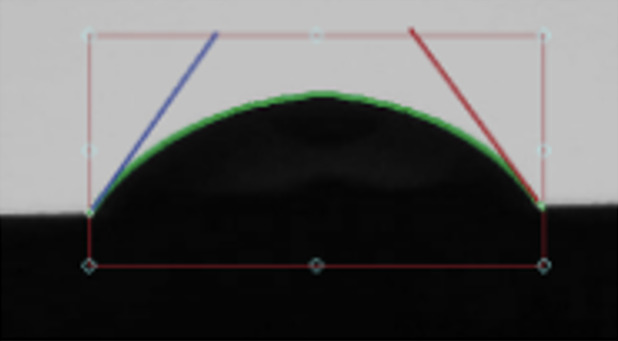

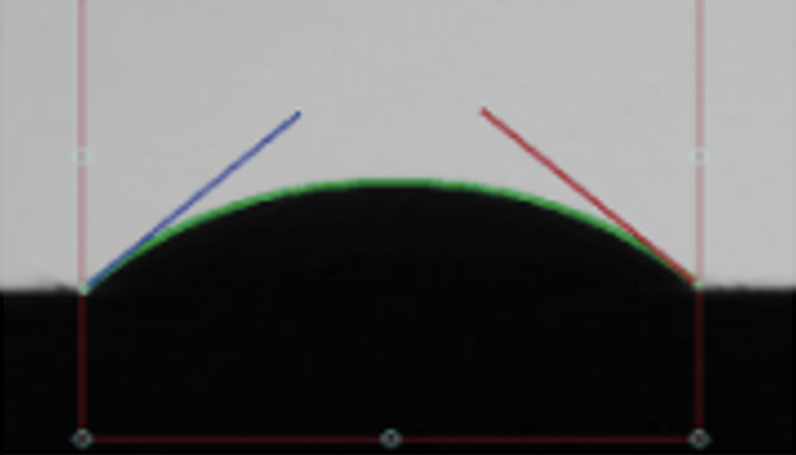

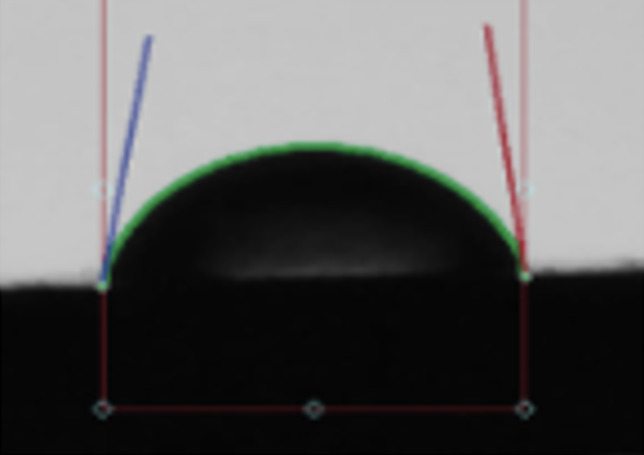
Depiction of contact angles of the titanium-SLA implant surface disk with (a) Peridex, (b) StellaLife, and (c) saline using the Ossila goniometer.

**Fig 2 fig2:**
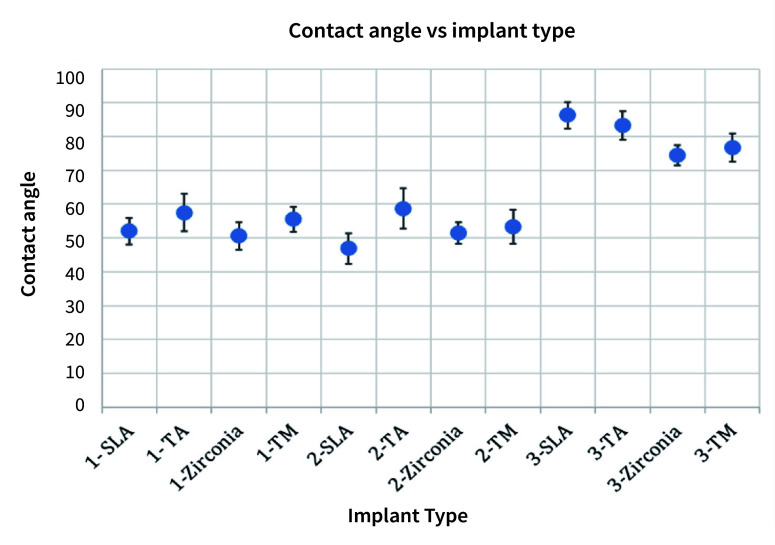
Contact angle measured for each implant type after dispensing each of the three liquids on the disks. For example, when zirconia was soaked with saline (3-Zirconia), the contact angle was found to be 74.5° with a standard deviation of 2.98. SLA: titanium-SLA; TM: titanium machined; TA: titanium alloy.

In group 1, the contact angles between CHX and SLA surfaces were found to be 52.0° ± 3.95 (Fig 1), between CHX and titanium alloy 57.5° ± 5.51, between CHX and zirconia 50.6° ± 4.14, and between CHX and machined titanium surface the contact angle was 55.5° ± 3.63 (Table 1 and Fig 2).

**Table 1 table1:** Mean contact angles between each liquid and implant surface along with the standard deviations

Implant type	Contact angles	SD
1-SLA	52.0°	3.95°
1-TA	57.5°	5.51°
1-Zirconia	50.6°	4.14°
1-TM	55.5°	3.63°
2-SLA	46.9°	4.50°
2-TA	58.7°	5.97°
2-Zirconia	51.5°	3.20°
2-TM	53.3°	5.11°
3-SLA	86.3°	3.89°
3-TA	83.3°	4.14°
3-Zirconia	74.5°	2.98°
3-TM	76.7°	4.19°
For example, when zirconia surfaces were irrigated with saline droplets (3-Zirconia), the contact angle was found to be 74.5°s with a standard deviation of 2.98°s. SLA: titanium-SLA; TM: titanium machined; TA: titanium alloy. SD: standard deviation.

In group 2, the contact angle between StellaLife and SLA was 46.9° ± 4.50 (Fig 1), between StellaLife and titanium alloy 58.7° ± 5.97, between StellaLife and zirconia 51.5° ± 3.20, and between StellaLife and machined titanium the contact angle was 53.3° ± 5.11 (Table 1 and Fig 2).

In group 3, the control group, the contact angles between saline and SLA surfaces were found to be 86.3° ± 3.89 (Fig 1), between saline and titanium alloy 83.3° ± 4.14, between saline and zirconia 74.5° ± 2.98, and between saline and machined titanium 76.7° ± 4.19 (Table 1 and Fig 2).

Statistical analysis with ANOVA showed that the mean difference between saline and Peridex as well as saline and StellaLife were statistically significant for all implant surfaces. The Bonferroni post-hoc comparison revealed a statistically significant difference with improved wettability for group 2 compared to groups 1 and 3 (p < 0.05) for rough-surfaced SLA implant surfaces. However, no statistically significant difference was seen between the other groups and implants (Table 2).

**Table 2 table2:** Bonferroni post-hoc comparison between the three liquids for each implant surface

Multiple comparisons, dependent variable: angle
Material	(I) group	(J) group	Mean difference (I-J)	Significance	95% Confidence Interval
Lower limit	Upper limit
TA	Saline	StellaLife	24.61*	<0.001	21.75	27.47
Peridex	25.83*	<0.001	22.97	28.69
StellaLife	Saline	-24.61*	<0.001	-27.47	-21.75
Peridex	1.22	0.906	-1.64	4.08
Peridex	Saline	-25.83*	<0.001	-28.69	-22.97
StellaLife	-1.22	0.906	-4.08	1.64
SLA	Saline	StellaLife	39.44*	<0.001	37.2	41.68
Peridex	34.26*	<0.001	32.02	36.5
StellaLife	Saline	-39.44*	<0.001	-41.68	-37.2
Peridex	-5.18	<0.001	-7.42	-2.94
Peridex	Saline	-34.26*	<0.001	-36.5	-32.02
StellaLife	5.18*	<0.001	2.94	7.42
Zirconia	Saline	StellaLife	22.95*	<0.001	21.06	24.84
Peridex	23.87*	<0.001	21.98	25.76
StellaLife	Saline	-22.95*	<0.001	-24.84	-21.06
Peridex	0.92	0.711	-0.96	2.89
Peridex	Saline	-23.87*	<0.001	-25.76	-21.98
StellaLife	-0.92	0.711	-2.81	0.96
TM	Saline	StellaLife	23.33	<0.001	20.97	25.69
Peridex	21.20*	<0.001	18.84	23.56
StellaLife	Saline	-23.33*	<0.001	-25.69	-20.97
Peridex	-2.13	0.092	-4.49	0.23
Peridex	Saline	-21.20*	<0.001	-23.56	-18.84
StellaLife	2.13	0.092	-0.23	4.49
*The mean difference is significant at the 0.05 level. ^§^For example, the mean difference between Peridex and StellaLife with zirconia surfaces was non-significant (p = 0.711). SLA: titanium-SLA; TM: titanium machined; TA: titanium alloy.

## DISCUSSION

The wettability between an implant surface and a liquid surface is affected by the implant’s surface roughness and the properties of the droplet. A synergistic effect has been determined between high surface roughness and surface hydrophilicity.^
6
^ Surface roughness is classified as smooth (Ra < 0.5 μm), minimally rough (Ra 0.5–1.0 μm), moderately rough (Ra 1.0–2.0 μm), and highly rough (Ra > 2.0 μm). Titanium SLA implants typically have a surface roughness of ~3.81 μm, indicating a highly rough surface,^
16
^ While zirconia implants have minimal roughness of ~0.66 μm. Other titanium implants have the least surface roughness with only ~0.59 μm.^
5
^


In this study, SLA implants had the lowest contact angle in group 2 and the second lowest in group 1. This suggests enhanced wettability with both CHX and StellaLife, with StellaLife exhibiting a more pronounced effect, which correlates to the better wettability provided by the herbal extract.

However, a statistically significant difference with improved wettability was only seen for group 2 (p < 0.05 vs groups 1 and 3) for the rough-surfaced SLA implant surfaces. Both titanium alloy and titanium machined were found to have the highest contact angles in groups 1 and 2, indicating a lower wettability, which correlates to the least surface roughness seen in machined surface titanium implants. CAs between zirconia disks were found to be in the intermediate range in group 2, which follows its minimal roughness. However, zirconia was found to have the lowest CA in group 1, which can be attributed to the benefits seen in CHX. No statistically significant difference was seen between zirconia, titanium alloy, or machined titanium, when any liquid was used to disinfect the disks.

The etiology of peri-implantitis, stemming from oral biofilms, can negatively affect wound healing and specifically peri-implant regeneration after surgery. Therefore, CHX is the rinse of choice for patients after implant surgery to reduce the risk of microbial adhesion and gingival inflammation.^
4
^ After two weeks of CHX use, there is a 50%-82% reduction in plaque.^
15
^ Nevertheless, chlorhexidine can alter the surface topography of dental implants and cause cell cytotoxicity. This can hinder the biological potential of osseointegration. Therefore, with the limitations of the present in-vitro study, the tested herbal rinse can be used as an alternative to CHX after implant surgery and in peri-implantitis cases.^
9
^


On the other hand, the ingredients in StellaLife show no harm to human cells.^
4
^ The ingredients of StellaLife include neem extracts, calendula, and chamomile. Neem can significantly reduce the release of proinflammatory cytokines, such as tumor necrosis factor (TNF-α), elevate the count of CD4+ and CD8+ T-cells, and inhibit nuclear factor-κB.^
11
^ Therefore, this herbal extract can demonstrate anti-inflammatory activities. Calendula exhibits antimicrobial properties, which can aid in wound healing. Additionally, a statistically significant reduction in plaque index, gingival index, and sulcus bleeding index was seen due to calendula. The chamomile ingredient assists in pain management due to its antinociception.

The data found in this study confirmed a previously published study showing that rougher implant surfaces are more hydrophilic than smoother surfaces.^
13
^ However, no data have been published comparing the effects of antimicrobial agents on wettability of implant surfaces, specifically herbal extract rinses. As seen in the results, titanium implants may have improved hydrophilicity when disinfected with herbal extracts. Hydrophilic surfaces increase cell attachment and osseointegration, and thus lead to implant success.

Ultimately, StellaLife in combination with hydrophilic and bioactive implant surfaces could provide a synergistic effect, promoting a more biocompatible environment around the implant. Reducing the bacterial load and infection risks without the adverse effects associated with chlorhexidine can shorten the recovery time for a patient. As a result, StellaLife may support favourable biological conditions required for improving clinical outcomes and patient comfort.

Limitations of this study include lack of clinical information, as the data were obtained using implant disks in-vitro. Further research should be conducted on various zirconia implant disks with different surface patterns, as only one type of zirconia disk was used in this study. Another limitation of the study was lack of investigation of how these agents affect tissues at a histological level. These changes might include alterations in cellular architecture, potential tissue damage, and inflammation. Without understanding these histologic effects, knowledge regarding the long-term impact is incomplete. Additionally, certain histological changes could influence the body’s immune response or healing processes, which might be crucial for treatment outcomes. A more comprehensive evaluation of the agents’ suitability and safety for therapeutic use is needed.

## CONCLUSION

Titanium implants may have improved hydrophilicity when rinsed with herbal extract antimicrobial agents compared to chlorhexidine.

## References
